# Operation for Strabismus Completed under Narcotism of the Bromide of Ethyl in Fifty-two Seconds for Ethylization and Tenotomy

**Published:** 1882-12

**Authors:** Julian J. Chisolm

**Affiliations:** Baltimore, Md.; Professor of Eye and Ear Surgery in the University of Maryland; Surgeon in charge of the Presbyterian Eye and Ear Charity Hospital, Etc.; 55 Franklin Street


*FOR CONTENTS OF THIS NUMBER SEE PAGE* 656.

*THE*

### Independent Practitioner.

VOL. III.——*December*, 1882.——NO. 12.

### ORIGINAL COMMUNICATIONS.

*We are not responsible for the opinions expressed by contributors.*

ARTICLE I.

### OPERATION FOR STRABISMUS COMPLETED UNDER NARCOTISM OF THE BROMIDE OF ETHYL IN FIFTY-TWO SECONDS FOR ETHYLIZATION AND TENOTOMY.

BY JULIAN J. CHISOLM, M. D., BALTIMORE, MD.

Professor of Eye and Ear Surgery in the University of Maryland ; Surgeon in charge of the Presbyterian Eye and Ear Charity Hospital, Etc.

Having within the past year performed many operations for the correction of strabismus under the quieting influence of the new anæsthetic, the Bromide of Ethyl, and being surprised more and more at the very short time required for producing deep sleep, during which the faulty muscle can be readily divided, I was desirous of knowing in how short a time the operation for the ugly condition known as crossed-eye or squint could be completed. I had repeatedly performed the operation before the medical class of the

University of Maryland, in what seemed to me to be within one minute, and my patients had seemingly been fully anæsthetized in apparently a very few seconds. To test the matter, the following case was operated on and time kept by stop watch :

Master W., a bright lad of 14 years, has had a permanent internal squint since he commenced school life, 8 years ago. The left eye has retained perfect vision ; but the right one, which is the squinting eye, can only make out large print, showing that in it, for want of use, there has been a deterioration of acuteness in vision. It is not on account of the dull sight that he seeks relief. His feelings are constantly ruffled by his rude school companions, and it is to escape being called cross-eyed, that he has applied to the Dispensary for operation. He has been told that he could be put to sleep and have his eyes straightened without pain, and without his knowing anything about it.

He loosened his collar, and got upon the operating table without hesitation, and laid down with his head upon a low pillow. In the meantime arrangements were being made for the operation. A towel was folded, and between one of the folds a thick piece of paper was placed. The towel was then folded into a cone with closed top, and secured in this form by a large pin. The paper makes the cone nearly air tight, and the space within the cone chamber gives free room for nose and mouth, both of which are to be covered in the administration of the anæsthetic. All the instruments necessary in performing the operation for squint were placed on the table within easy reach, and in the order in which they would be used. They were the wire stop speculum for keeping the eye-lids separated, forceps for seizing the conjunctiva, scissors for cutting the tissues, and hook for securing the tendon to be divided ; also a piece of soft sponge which had been previously wetted for keeping the wound clean of blood. The lad was now told that when the towel was placed over his face he would feel a little choked, but that the quickest way to get over that feeling was to take long breaths. He was made to go through the performance with the dry towel over his nose. An assistant, who had often given the Bromide of Ethyl for me, and who was familiar with all the steps of the operation for squint, stood at the head of the table.

All being in readiness, including time-keeper with stop-watch in hand, about a drachm of the Bromide of Ethyl was poured into the cone, and immediately inserted over the mouth and nose of the patient, and with rim of the cone held down firmly upon the face so that very little air could enter. The object was to make as much as possible a saturated ethyl atmosphere shut up in the cone for the patient to breath. Without much resistance the patient commenced to breathe quickly. After a very few inspirations the breathing became slow and regular, pulse remained unchanged, the natural color remained in his face and ears, and he seemed to be in a deep sleep. The conjunctiva, the last surface to lose reflex action, was touched and gave no evidence of irritation. Time was called and reported 22 seconds from the moment of putting the cone over the face to the stage of deep ethyl narcosis.

The cone was now removed and the operation commenced without any loss of time. The wire speculum was rapidly placed in position. A fold of conjunctiva corresponding in position to the lower border of the internal rectus muscle was seized and divided horizontally, so as to make an incision which reached from near the border of the cornea to the vicinity of the caruncula. As this little wound gaped, the exposed fascia was in like manner divided by the scissors. The hook was then introduced into this hole, the tendon of the internal rectus muscle seized, drawn forward, exposed in the wound by the gaping of the conjunctival opening, and dissected away by scissors clipping, from its sclerotic attachment. A second introduction of the hook proved the tendon to have been completely divided throughout all its wide attachment to the eye ball. The hook was then withdrawn, speculum removed, and the operation was completed. Time was again called. *It was found that just 30 seconds had been occupied in the various steps of the operative manual, making in all 52 seconds from the moment that the cone was placed over the face, to the removal of the speculum holding apart the lids.*

The blood oozing from the conjunctival wound had only been wiped away, when the lad awoke, sat up on the table, and then jumped to the floor. *His ethylized sleep had not exceeded two minutes at the very outside.* He awoke as from an ordinary sleep, with clear brain, no nausea, no headache, no lassitude, no giddiness, in fact, feeling quite as well as if he had taken no anesthetic.

Fifty-two seconds for the ethylization and squint operation is the nearest approach to the miraculous, and yet can be accomplished by any surgeon familiar with the handling of eye instruments, and who will become personally acquainted with the wonderful virtue of the new anæsthetic.

Bromide of Ethyl can never replace chloroform or sulphuric ether. Its effects pass off too quickly for slow operators or for tedious operations; and although it can be again and again inhaled, the reproduction of anæsthesia is most apt to be followed by the nausea, vomiting and depression of chloroform or ether.

*For what I will call the primary anæsthetic sleep, from the first inhalation of the Bromide of Ethyl,* I know nothing in the list of anesthetic agents that can compare with it, in the rapidity of its action, the fullness of the narcotism produced, and the very evanescent character of the sleep, which, although deep, did not continue in any of my patients now numbering fully 400 cases, more than two minutes. At the same time I have so far found no patient who can resist its sleepy influence.

Twenty to sixty seconds in every case in my experience has been sufficient to induce deep narcotism, provided I made the patient inhale a drachm of the Bromide of Ethyl in a nearly air tight cone, so that the atmosphere should be a saturated one. Once the cone is put over the face, and held firmly down, it must not be removed, notwithstanding the struggling of the patient, till deep narcotism is obtained, when his conjunctiva will show no reflex irritation on touching it. The conjunctiva being the most sensitive surface in the body, the absence of reflex irritation in it, under anæsthetic narcoses, always means the complete abolition of sensation in the whole body. Sometimes I have obtained this deep sleep by the 6th or 8th inhalation. If air is allowed to enter the cone freely I am not sure that the patient will go to sleep at all; and after the breathing of much ethyl in futile attempts to procure narcosis, intense and long continued nausea is very apt to be induced.

I am one of those who believe it to be the province of modern surgery to do all possible good with the least amount of pain, and with as little risk as possible. The amount of good obtained from chloroform and ether in saving lives, as well as destroying pain, who can calculate? Of late the tendency has been with the theorists in therapeutical agents to decry the fatality of these most valuable remedies, accidents of the rarest occurrence, when contrasted with their everyday use and daily distribution of blessings. Since their introduction into surgical practice 35 years ago, there has been a constant desire to find some agent, which will do all the good that chloroform or ether dispenses, and at the same time, not expose patients to the dangers which sometimes, however rarely, follow in the wake of their administration. Have we discovered such an agent in Bromide of Ethyl ? My own experience in now over 400 inhalations, looks as if we have found the much desired agent, for at least a very large number of cases in which the older anæsthetics have heretofore been used.

*I believe if the Bromide of Ethyl be given in a concentrated form, it will always produce deep*, although not long lasting sleep. During this very temporary narcosis any painful surgical operation which should only occupy a minute of time can be safely and painlessly completed. For such operations I use it daily, and with a growing confidence.

I commend this new anesthetic as the most prompt, most evanescent, least nauseating and by far the most valuable of all sleep producing agents for all quick surgical work. Any surgeon, who has seen it used once, will ever afterwards consider it as among his most valuable agents.

55 Franklin Street.

